# Dynamics of entomopathogenic nematode foraging and infectivity in microgravity

**DOI:** 10.1038/s41526-020-00110-y

**Published:** 2020-08-10

**Authors:** Fatma Kaplan, David Shapiro-Ilan, Karl Cameron Schiller

**Affiliations:** 1 Pheronym, Inc., Davis, CA 95618 USA; 2 US Department of Agriculture, Agricultural Research Service, Byron, GA 31008 USA

**Keywords:** Microbial ecology, Microbial ecology

## Abstract

Microgravity is a unique environment to elucidate host–parasite biology. Entomopathogenic nematodes (EPNs), model parasites, kill host insects with mutualistic bacteria and provide environmentally friendly pest control. It is unknown how microgravity affects a multistep insect invasion by parasites with mutualistic bacteria. EPNs respond directionally to electromagnetic cues and their sinusoidal locomotion is affected by various physical factors. Therefore, we expected microgravity to impact EPN functionality. Microgravity experiments during space flight on the International Space Station (ISS) indicated that EPNs successfully emerged from consumed insect host cadavers, moved through soil, found and infected bait insects in a manner equivalent to Earth controls. However, nematodes that developed entirely in space, from the egg stage, died upon return to Earth, unlike controls in microgravity and on Earth. This agricultural biocontrol experiment in space gives insight to long-term space flight for symbiotic organisms, parasite biology, and the potential for sustainable crop protection in space.

## Introduction

Entomopathogenic nematodes (EPNs) in the genera *Heterorhabditis* and *Steinernema* are insect parasites used as biocontrol organisms in eco-friendly agricultural pest control as well as model organisms for parasite biology^1–4^. The EPN life cycle has two main phases: a free-living phase in the soil and a parasitic phase inside the insect. Infective juveniles (IJs) are the only stage that is free-living in the soil and can survive for months without food^1–3^. Anatomical and physiological changes in this stage include the cessation of feeding, closed mouth and anus, the presence of a double cuticle layer and resistance to stressful environmental conditions. IJs kill their insect hosts with the aid of symbiotic bacteria carried in the nematode gut (*Photorhabdus* spp. bacteria are associated with *Heterorhabditis* spp. and *Xenorhabdus* spp. bacteria are associated with *Steinernema* spp.)^2,5,6^. The parasitic phase begins when IJs enter insect hosts through natural openings (mouth, anus, and spiracles), or occasionally through the cuticle. The nematodes then release their mutualistic symbiotic bacteria, which reproduce and help bring about host death by septicemia or toxemia within 24–48 h^7–9^. Nematodes also contribute with their own toxins and immune suppressors^10^. Within the host, the nematodes undergo normal development consisting of four juvenile stages (J1–J4) separated by four molts. The final molt results in the reproductive adult stages. Nematodes carry out one to three generations within a single host over a 10–22-day period^2,5^. When nutritional quality declines and waste products increase, the specialized third-stage juvenile, the IJ, is formed. The IJs then emerge from the insect cadaver and proceed to seek the next host in the soil environment^2,3,11^.

Foraging and infection are critical life-cycle steps for most parasites. Foraging is finding a host and infection is accepting and successfully establishing in a host. EPN foraging and infection are influenced by various factors inside and outside the host cadaver^3,12–15^. For example, nematode dispersal to find hosts depends on sinusoidal locomotion on wet surfaces (e.g., soil), which is governed by certain physical properties under a gravitational field^16,17^. Additionally, some parasitic nematodes, including EPNs, are thought to navigate in part based on electromagnetic fields^18^. These physical factors that impact locomotion and navigation would be absent or altered under microgravity conditions. Thus, studying EPN movement and infectivity under microgravity conditions could shed light on the relative importance of such factors as they contribute to nematode foraging success on Earth. Moreover, it is of interest to determine the impact of microgravity on the subsequent stages of the EPN life cycle including pathogenesis, host-immune response, symbiotic interactions, and reproduction. Thus, our objective was to study EPN foraging and infection dynamics in space.

Understanding foraging and infectivity in space is critical to studying space biology of parasites in general. The International Space Station (ISS) is a unique environment^19^ to study space biology. Inside the ISS, the temperature ranges between 21 and 23 °C. Organisms are exposed to ionizing radiation from galactic cosmic rays (energetic particles from outside our solar system), particles trapped in the Earth’s magnetic field (the Van Allen Belts), solar energetic particle events (solar flares), and microgravity where gravitational loading, hydrostatic pressure, convection, buoyancy, and sedimentation do not exist^19^. Investigating the efficacy of natural biocontrol agents such as EPNs at ISS could help establish successful agriculture and plant protection in space because growing plants in space is important for bioregenerative life support systems during long-term human space flights^20–27^. As expected, many aspects of plant physiology, growth and development were extensively studied both at ISS and on Earth including response to high CO_2_ levels^20,21^, seed development^25–27^, and spaced-induced hypoxia^23,24^. Microgravity itself is a unique physical factor that causes many other environmental factors to behave differently. For example, water behaves very differently in microgravity in space *versus* on Earth^28^. EPN IJs survive within water films in interstitial spaces in soil, where factors such as moisture and soil type affect survival and dispersal^3,16^. Predicting the outcomes of an agricultural biocontrol agent in microgravity is difficult because many environmental factors are affected by microgravity (water behavior, lack of buoyancy-driven convection), in addition to required cooperation of two organism to execute a multistep infection. To the best of our knowledge this is the first agricultural biocontrol experiment in space.

## Results

### EPNs IJs in microgravity can emerge, disperse, forage in sand, invade a healthy insect, develop, and reproduce

**Fig. 1:**
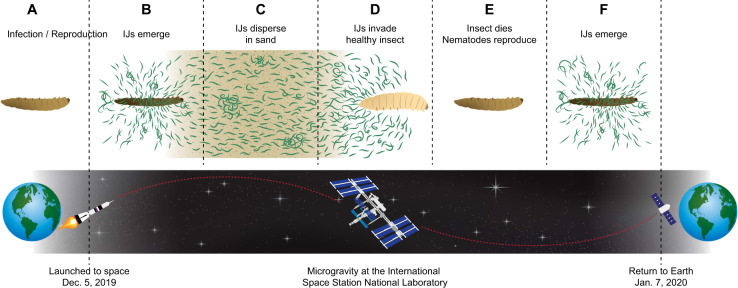
*Steinernema feltiae* IJ emergence, host invasion, and reproduction in microgravity. Panels (**a**–**f**) were tested with four concurrent experiments called as Specimens. **a**–**d** Specimen 1. **a**–**f** Specimen 2. **a**–**c** Specimen 3. **c**–**f** Specimen 4.

**Fig. 2:**
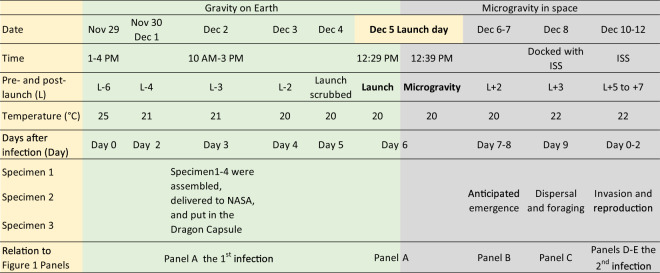
Experimental timeline from specimen preparation on Earth to docking with ISS. Yellow backround indicates the data contained in each row, green background indicates gravity on Earth, and gray background indicates microgravity in space. The bottom panels show the relationship of the Figure 1 panels with timelines.

To determine whether EPN IJs in microgravity (Figs. 1, 2) can emerge, disperse, forage in sand, invade healthy insect larvae and then complete a reproductive cycle, we designed four experiments (details of the design in method section) which were run concurrently on the ISS U.S. National Laboratory (NL) and on Earth. The result of the first experiment (Specimen 1), depicted in Table 1, indicate that IJs were able to emerge from the consumed insect host cadaver in microgravity (Figs. 1b, 2), travel through 10 cm of moist sand (Figs. 1c, 2) and invade a healthy bait insect host (Fig. 1d). No difference in IJ invasion was detected between the nematodes from the space station and their Earth controls (*t* = −0.42; df = 10; *P* = 0.68) suggesting that microgravity did not affect host invasion. Furthermore, host-immune response to IJ invasion, based on hemocyte encapsulation, in microgravity (Fig. 1d) was not different from the Earth controls (*t* = −0.02; df = 10; *P* = 0.99) (Table 1). Also, some of the nematodes inside the bait insect in Specimen 1 (Fig. 1d) were IJs and some became adults (IJ–J4-adult), suggesting that they could recover and continue development in microgravity. However, Specimen 1 was frozen while on the ISS before the IJs could reach sexual maturity and reproduce (Fig. 1e–f).

**Table 1:** Specimen 1 *Steinernema feltiae* IJ host invasion and host-immune response in Fig. 1d.

Treatment	Rep (*n*)	Bait insects	Infected insects	Invading IJ (# ± sem)	*P* value	Hemocytes (# ±sem)	*P* value
Space (Microgravity)	3	6	6	6.2 ± 2.4	0.68	2.5 ± 1.3	0.99
Earth (Control)	3	6	5	6.5 ± 3.1		2.3 ± 1.1	

To determine whether EPNs reproduce in microgravity from eggs (Fig. 1e–f), we analyzed a concurrent experiment, Specimen 2, which was allowed to develop in the bait insect host and reproduce in microgravity. Consistent with Specimen 1, the IJs in Specimen 2 were able to recover or resume development and turn into adults. The adults in the bait insect developed eggs, reproduced, and their progeny turned into IJs and emerged (Fig. 1f) in microgravity (Table 2). The IJs in Fig. 1f are from the second infection, which occurred in microgravity during this experiment. Only one of three replications produced IJs. Corresponding Earth controls were then examined to determine whether the reduced development was due to microgravity. The Earth control had the same ratio of emergence (Table 2), i.e., only one of the three replicates emerged, suggesting that the reduced emergence was not due to microgravity. Specimens 1 and 2 together suggested that EPNs can go through both phases of their life cycle, free living and parasitic inside the host, in microgravity. They can enter an insect host, release their symbiotic bacteria which, along with nematodes, kill the insect and the EPNs feed and reproduce. In both specimens 1 and 2, the IJs had to forage (at least 10 cm) to invade a host, which provides indirect evidence that *S. feltiae* IJs emerged from the consumed host cadavers in microgravity. However, the direct evidence is provided by Specimen 3 where IJs from the first infection emerged into sand in microgravity without a bait insect to invade. The sand was used as a trap to store the IJs. Similar to Specimen 2, Specimen 3 had only one replication out of three emerge (Table 2) and the corresponding Earth control did not have any emerged IJs.

**Table 2:** *Steinernema feltiae* IJ adaptation to Earth’s gravity after microgravity in Fig. 1b/c (Specimen 3) and f (Specimen 2) and their symbiotic bacteria.

Treatment	*n*	Eggs formed	Emerged location, rep (*n*)	IJs return to Earth	*n*	IJ disperse (%)	*P* value	*n*	Bacteria colony/IJ (# ± sem)	*P* value
Specimen 2 Space (Microgravity)	3	Space	Space (1)	All dead				10	2.3 ± 1.0 a	0.0001
Earth (Control)	3	Earth	Earth (1)	Alive	3	39 ± 7.9	0.86	10	153 ± 29 b	
Specimen 3 Space (Microgravity)	3	Earth	Space (1)	Alive	3	41 ± 5.5		10	219 ± 35 c	0.04
Earth (Control)	3	Earth	Earth (0)	NA				10*	138 ± 37* b	

*n* replication number.

*Lab standard.

### IJs reproduced in microgravity from eggs could not adapt to Earth’s gravity

**Fig. 3:**
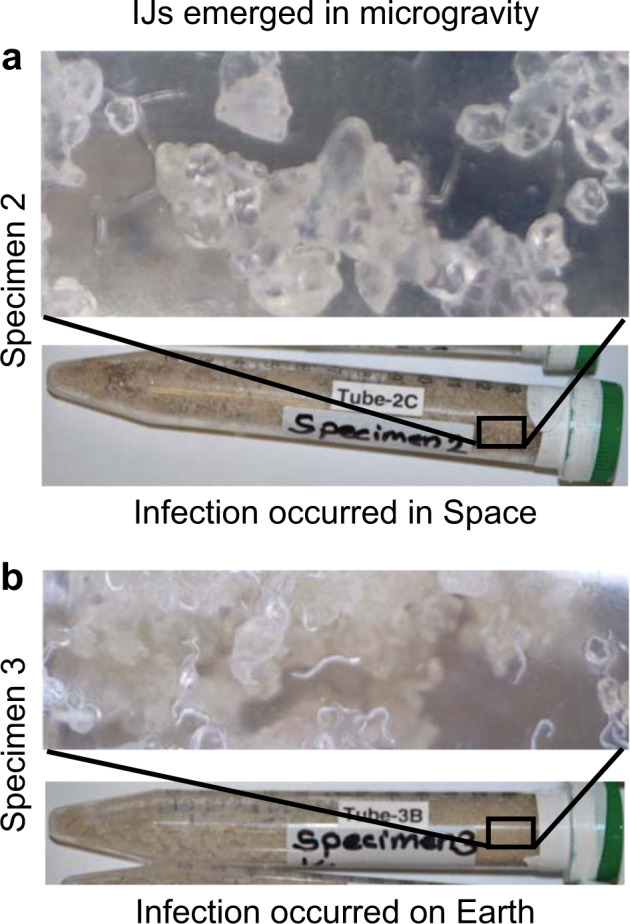
*Steinernema feltiae* infective juveniles (IJs) in Specimens 2 and 3 after returning to Earth. **a** Specimen 2 IJs observed three days after returning to Earth. IJ tails seemed to be bent. These IJs were from the second infection, the bait insect infection, in microgravity. They were estimated to travel in space for ~20–23 days after emergence from consumed cadavers. **b** IJs from the first infection in Specimen 3 were observed 3 days after returning to Earth. The IJs displayed sinusoidal movement and seemed to adjust to Earth’s gravity. These IJs emerged from a consumed host in microgravity and traveled ~27–30 days in space. The host was infected on Earth, meaning part of their development was on Earth before they formed IJs. Figures in both **a** and **b** are showing the one replication which emerged out of the three replications from each of the Specimens. Crystals in the figures are sand.

To our surprise, the Specimen 2 IJs were all dead and bent when they arrived on Earth (Fig. 3). Our first thought was that maybe Specimen 2 ran out of air and died, so we examined the Specimen 2 Earth control which contained live and active IJs (Table 2) suggesting that the Specimen 2 in space did not run out of air. However, the physical environment of their space treatments was more dynamic and differed from the Earth controls as the free volume of the interstitial spaces and distribution of water and oxygen would be different given the absence of gravity in the space treatments. The Earth control did not rule out the possibility of space-induced hypoxia which is a well-known phenomenon in plants^23^. To determine whether this was space induced hypoxia, we examined Specimen 3, where IJs from the first infection were allowed to emerge in space and trapped in sand in microgravity (Fig. 1b, c) with no bait insects. The IJs in Specimen 3 were alive and showed sinusoidal movement after returning to Earth (Fig. 3b) suggesting that the IJs’ deaths may not have been due to just space-induced hypoxia.

Alternatively, the IJs produced from the second infection in Specimen 2 could not adjust to Earth’s gravity. The IJs in Specimens 2 and 3 all emerged in space (Table 2) and traveled in space. The major difference between the two specimens were IJs in Specimen 2 (Fig. 1d–f) completely developed in space starting from egg stage (egg-J1-J2-IJ) and IJs in Specimen 3 (Fig. 1a–c) started their development on Earth and completed it in space. So, the exposure to gravity during development may play a role in adaptation to Earth’s gravity. Therefore, we examined the 4th concurrent experiment (Specimen 4) for adaptation of the IJs developed in space to Earth’s gravity. The IJs produced in this experiment would be comparable to IJs in Specimen 2. Unfortunately, none of the replications in Specimen 4 reproduced in microgravity or on Earth. We tested whether Specimen 4 IJs were infective when they returned to Earth and found that one replication from both microgravity and the Earth control infected bait insects, suggesting that IJs were capable of invasion. Even though each replication in Specimens 2 and 3 produced populations of nematodes (~4000 IJs/conical tube harvested), it is difficult to make a conclusion because only one replication from Specimen 2 reproduced and all the IJs died when they returned to Earth, and one replication from Specimen 3 reproduced and the IJs survived. Therefore, we examined Specimen 5 to see whether EPN IJs can adapt to Earth’s gravity. Instead of sand, Specimen 5 was filled with a polyacrylamide gel and contained the starter IJs, which were used to conduct the first infections of the specimens 1, 2, and 3. All three replications of Specimen 5 successfully traveled in space for 33 days and adapted to Earth gravity after return (Supplementary Video 1 and 2). The experiments in microgravity and their corresponding Earth controls in Specimen 2, 3, and 5 suggest that the timing of EPN development, particularly in reference to egg and/or J1 formation, impacts the nematode’s ability to adapt to gravity such as when returning to Earth, and requires further study.

### Symbiotic bacteria loading during IJ formation may differ in microgravity, but dispersal was not affected

We next asked whether the adaptation to Earth’s gravity was the only difference in the IJs in Table 2. We first looked at IJ dispersal because it requires movement and muscle power. Since the IJs from the second infection in Specimen 2 that developed in microgravity were dead upon return to Earth, dispersal was not tested. IJs from the first infection in Specimen 3, which emerged in microgravity, showed 41% dispersal (Table 2). Unfortunately, none of the replications of Specimen 3’s Earth control produced IJs. Therefore, we compared dispersal of Specimen 3 to Specimen 2’s Earth control (a good proxy control) which showed 39% dispersal. There was no significant difference (*t* = −0.19; df = 4; *P* = 0.86) in IJs’ dispersal between IJs emerged in microgravity or that of on Earth (Table 2).

We examined the symbiotic bacteria load per IJ as this may contribute to EPN fitness. Having more symbionts/IJ has a trade-off with reduced life expectancy^29^. There was a significant difference in symbiotic bacteria colony numbers between IJs emerged in microgravity and IJs emerged on Earth (Table 2). Specimen 2 IJs from the second infection in microgravity had significantly (*t* = 10.81; df = 18; *P* < 0.0001) fewer bacterial colonies compared to their Earth control. In contrast, Specimen 3 IJs from the first infection had significantly higher (*t* = −2.2; df = 18; *P* = 0.04) symbiotic bacteria compared to an Earth laboratory standard. Given that none of the Earth controls for Specimen 3 emerged, we used a laboratory standard which was not different from Specimen 2’s Earth control in terms of bacterial colony/IJs for comparison. Specimens 2 and 3 suggested that depending on time of development, microgravity may have an effect on IJ fitness upon return to gravitational environment.

### IJ space flight does not affect infectivity

The first four experiments investigated EPN infection and development in space but did not address whether IJ infectivity (host invasion) was affected after a prolonged (33-day) microgravity exposure. Specimen 5 was prepared in a concurrent experiment where *S. feltiae* IJs were stored in polyacrylamide gel, a different medium from the first four specimens, and were exposed to space flight on the ISS for 33 days. Upon return to Earth, we examined whether they were alive, sluggish or dead. Luckily, all three replications were alive along with their Earth controls (Supplementary Video 1 and 2). The infectivity tests were conducted at the earliest time that was logistically possible (10 days after returning Earth). The data in Table 3 suggest that there was no difference in space flight-exposed IJs infectivity compared to the Earth-bound controls (*t* = 0.23; df = 28; *P* = 0.82) (Table 3).

**Table 3:** Specimen 5 *Steinernema feltiae* IJ movement and infectivity after 33-day Space Flight.

Treatment	Rep (*n*)	IJs^a^	Movement^a^	Rep (*n*)	Infected insects	Invading IJ (# ± sem)	*P* value
Space (Microgravity)	3	Alive	Active	15	14	2.7 ± 0.6	0.82
Earth (Control)	3	Alive	Resting	15	13	3.0 ± 0.6	

^a^Evaluated 3 days after returning to Earth.

## Discussion

Our study revealed the microgravity effects on the pathogenesis and development of two mutualistic organisms; nematodes and bacteria. Based on Specimens 1, 2, and 3, *S. feltiae* can retain its symbiotic bacteria in microgravity, travel 10 cm through moist soil, infect bait insects, and complete at least one two-generational cycle. This is consistent with the space biology of the closely related model nematode *Caenorhabditis elegans*, which can complete at least two-generational cycles in microgravity^30–34^.

Adaptation of nematodes to Earth’s gravity upon return yielded surprising results. *C. elegans* dauer stage, analogous to EPNs IJs stage, are so resilient that they endured a space shuttle disintegration during re-entry to Earth and survived^35^. These *C. elegans* dauers that formed during the space flight and spent time in space, were alive upon return to Earth^35^. Therefore, we targeted the EPN IJs stage for further analysis after returning to Earth at ambient temperature. Furthermore, the IJ is the only free-living form in soil, making it easy to collect synchronized animals. Similar to *C. elegans* dauers, Specimen 3 IJs, which developed during the first 4 days of flight, returned to Earth alive. IJs in Specimen 5, which only traveled in space without infecting hosts, were also alive and adjusted to Earth’s gravity.

**Fig. 4:**
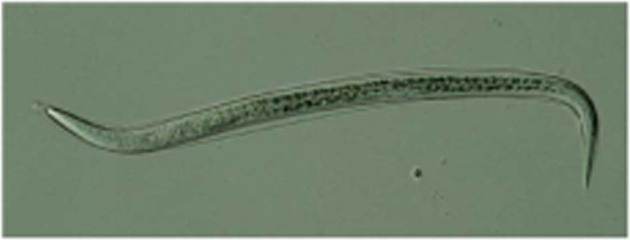
An EPN infective juvenile, *Steinernema carpocapsae*, with a bent or “J” shaped tail during rest or stress. Photograph used with permission from Bugs for Growers^48^.

To our surprise, IJs from the second infection in microgravity in Specimen 2 were all bent and dead. The difference between Specimens 2 and 3 was that the IJs in Specimen 3 emerged from cadavers infected on Earth whereas the IJs in Specimen 2 emerged from cadavers infected in space and therefore their development from the egg stage to IJ occurred entirely in microgravity. Thus, the nematodes in Specimen 2 were not exposed to gravity during their development. One explanation for the IJ death in Specimen 2 is space-induced hypoxia. Additionally, space-flight alters hypoxia related gene expression in nematode *C. elegans*^36^, but on Earth hypoxia extends *C. elegans* lifespan in the right conditions^37^. EPNs go into a bent tail posture when stressed^38^ or resting (Fig. 4). Based on the appearance of the IJs, we suspect the IJs became stressed upon re-entry to Earth’s gravity due to the pressure of being exposed to gravity, went into their bent tail posture, and then died.

What may be causing stress during re-entry? Our data in Specimens 2 and 3 suggest that the timing of EPN development, particularly in reference to egg formation, critically impacts their ability to survive a return to gravity and needs to be studied further to understand the molecular mechanisms involved. This can be achieved by cryopreserving EPNs while they are on the ISS^39^ for analysis after return. This issue is important for long-duration space flights of nematodes to destinations such as Mars. Nematodes may need to be transported as dauers/IJs to ensure they survive on their destination planet. Alternatively, if the IJ stage cannot survive all the way to Mars due to depletion of energy reserves then they need to be cryopreserved all the way to the other planets or placed into the parasitic phase (by infecting insects) to obtain nutrition and reproduce. If the latter is the case, the following questions need to be answered: Do we need to have them exposed to gravity, simulated or otherwise, during space flight? For how long and at which developmental stage? How does space-induced hypoxia affect EPN IJ development and fitness in space?

Differential survival in response to Earth’s gravity was not the only difference in IJs that developed in microgravity. Bacterial count/IJs in Specimen 3 was higher in the treatment (space) nematodes relative to their controls. It is not clear what caused an increase in symbiotic load in Specimen 3; conceivably, some trade-offs present on Earth are diminished in space^29,40^. The higher bacterial loading observed in Specimen 3 and its trade-offs should be investigated further in microgravity to determine whether there is an advantage for space flight or infectivity. In contrast, bacterial count/IJs in Specimen 2 was lower compared with their Earth controls. The lower bacterial count/IJ in Specimen 2 could be due to IJ death upon return to Earth (and subsequent deterioration of the symbionts). The observations from Specimens 2 and 3 gave a glimpse of how EPN development in microgravity may affect their adaptation to Earth’s or Mars’ gravity and their relationship with their symbiotic bacteria during space flight. Since Specimens 2 and 3 each had one replication emerge with a population of IJs (and despite using multiple IJ replicates in our experiment), these findings require additional replications to make conclusions. Furthermore, symbiotic bacterial load, which is important to nematode fitness, was also impacted, further emphasizing the need to study how microgravity affects EPNs’ adaptation to Earth’s gravity.

Studying EPNs in microgravity has positive implications for both basic and applied sciences as well as the potential for near-term practical application. EPNs, as model parasites, provide an opportunity to study parasite biology in space and complement *C. elegans* as a model organism to study space biology^30^. Unlike *C. elegans*, EPNs are pathogenic and have a specialized relationship with their bacteria. These two systems together can facilitate a variety of studies applicable to a broad range of disciplines, which neither can do individually. For example, by making comparisons between EPNs and *C. elegans*, we can apply results of *C. elegans* space experiments to fundamental questions of pathogenesis and symbiosis. Additionally, EPNs are part of a healthy soil ecosystem, provide eco-friendly pest control solutions, and offer a unique opportunity to establish agriculture for future space exploration and colonization. Insights from studying EPNs in space may also provide tools to improve the efficacy of EPNs as biological control agents for Earth applications.

## Materials and methods

### Rearing EPNs

*Steinernema feltiae* (SN strain) IJs were obtained from the International Entomopathogenic Nematode Collection (USDA-ARS, Byron Georgia USA). The nematodes were cultured in the laboratory using the White trap method^41^. *Galleria mellonella* (Vanderhorst Wholesale Inc. St. Marys, OH) were exposed to 100 IJs per larva. Infected *G. mellonella* larvae were incubated for 4 days at RT (20 ± 1 °C) and insect cadavers were transferred to White traps for IJ collection.

### Why *Steinernema feltiae* was chosen

*S. feltiae* is a widely studied nematode and commercially used as a biocontrol agent in diverse systems. We selected *S. feltiae* for two reasons. (1) The dispersal behavior at the expected ISS temperature was the main factor in our decision. *S. feltiae* disperses without a quiescent period at temperatures from 15 °C to 30 °C. Some other EPNs such as *S. carpocapsae* IJs disperse normally at or above 25 °C, but at 20 °C and below, the nematodes have a quiescent period where IJs stay stand still for period of 40 min to 24h^14^. ISS ambient temperature is between 21 and 23 °C. We did not want to take chance with potential failure due to a temperature fluctuation so we chose *S. feltiae*, which is known to be active at the temperatures^42^ of the ISS. (2) *S. feltiae* is an intermediate forager thus incorporating behaviors of both foraging extremes^43^.

### NASA safety certification, flight configuration, and safety experiments

**Fig. 5:**
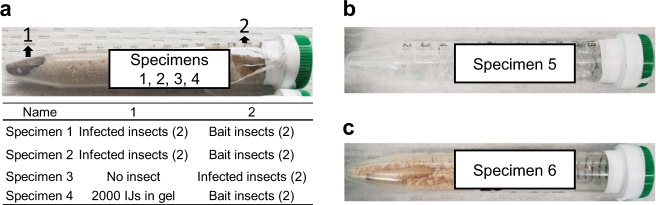
Experimental design of specimens for microgravity and their Earth control. **b** Specimens 1–4. One end of the tube, designated as 1, had either two infected insect hosts for providing infective juvenile nematodes (*Steinernema feltiae*) or 2000 IJs in 0.5 ml of polyacrylamide gel. The other end of the tube, designated as 2, had either two healthy bait insects or infected insect. **b***S. feltiae* IJs in gel. 4000 IJs/ml of polyacrylamide gel. **c** Two healthy *Galleria mellonella* larvae with wood shavings as shown.

The NASA safety certification was conducted by an implementation partner, NanoRacks, LLC (Houston, TX). The safety certification included filling out safety and experimental plan documents, submitting a bill of materials, and providing material safety sheets for the materials that would be flown to the ISS, their MSDS, and conducting safety and flight configuration experiments. To determine the risk of freezing tubes with moist sand on the ISS cold stowage, we sent three 15 ml conical tubes filled with 10% moist sand to NanoRacks for NASA safety testing. Briefly, play sand (Quikrete, Atlanta, GA) was washed three times in tap water and rinsed three times with MILLIQ water (MILLIPORE, Burlington, MA) using a gold pan (Garrett’s gravity trap, Garland, TX) then dried at 60 °C in the oven for 3 days or until all the moisture was evaporated. In a separate dish, dry sand was moistened with 10% MILLIQ water (w/v). Twelve ml moist sand was placed in 15 ml conical tubes that were rinsed three times with MILLIQ water and dried prior to use. We sent three tubes with moist sand to NanoRacks to be tested at NASA for safety. Next, we moved forward with flight configuration experiments for Specimens 2–6, prepared as shown in Fig. 5. Specimens 2–6 each with two replications were conducted once at 23 ± 2 °C and once at 15 ± 1 °C to determine whether nematodes can emerge and go through in the sand, infect the bait insects and reproduce within a month in a horizontal position. At the same time, we examined whether the tubes for Specimens 2–6 developed any discoloration or warp due to the byproducts produced during infection and decomposition of the cadavers for a month. Later, the flight configuration experiment was extended to six months in case return to Earth would be delayed due to lack of space in the resupply capsule. The lower temperature (15 °C) was tested as a precaution to determine whether nematodes still infect if the temperature fluctuated unexpectedly at ISS. We did one last safety test for Specimen 6 (insect only) to determine whether gasses released by decomposing insects could discolor or warp the tubes, causing a hazard on the ISS. For this experiment, two *G. mellonella* prepared in Fig. 5c were first placed in −80 °C overnight to kill insect larvae humanely and then the tubes incubated at 23 ± 2 °C. Two replications with a total four insect larvae were monitored monthly for over 6 months for discoloration and warping due to decomposing insect larvae. No warping or discoloration was observed, fulfilling safety requirements to move forward with the experiments.

### Preparing IJs for the microgravity experiments

*S. feltiae* IJs were removed from the White trap 4 days after the beginning of emergence and rinsed three times in deionized water to remove residual cadaver-derived metabolites and pheromones (Supplementary Table 1) as described by Kaplan et al.^14^. Rinsed IJs were resuspended to infect the *Galleria mellonella* larvae for Specimens 1, 2, and 3 and used to prepare IJs in polyacrylamide gel (75 ml of water 1 g of a polyacrylamide gel (Soil Moist, JRM Chemical, Cleveland OH) with a density of 4000 IJs/ml for Specimens 4 and 5 as shown in Fig. 5a for both microgravity experiments and their corresponding Earth controls.

### Six concurrent experiments in microgravity for emergence, infection and reproduction and space flight

We designed multiple concurrent experiments that were conducted at the International Space Station U.S. National Laboratory (ISS) with controls conducted on Earth. The experiments were designated as Specimens 1–6 (Fig. 5). The concurrent experiments were conducted to capture the maximum amount of information about the EPN infection process and life cycle in microgravity and make sure that usable data was returned even if microgravity interrupted part of the EPN life cycle. The space limitations of conducting an experiment on the ISS also had to be considered. Microgravity causes changes in many variables (not just gravity), which may impact a multistep infection requiring the cooperation of two organisms (nematode and its mutualistic bacteria). Furthermore, some of the facilities on the ISS, such as cold stowage (−80 °C), have limited capacity and high demand. A timeline of the experiments (Specimens) is presented in Fig. 2 and Supplementary Table 1.

We used a modified sand column assay^44,45^ shown in Fig. 5. The experimental units consisted of 15 ml conical tubes (Fig. 5) filled with *S. feltiae* infected *G. mellonella* larvae or 2000 *S. feltiae* IJs in 0.5 ml of polyacrylamide gel were placed in one end of the tube and the healthy larvae were placed on the other end of the tube (CELLTREAT scientific products, San Diego, CA). Based on preliminary evidence, the set-up allows a 3-day time delay for the nematodes to move through the sand and infect the bait larvae. However, we did not know what delay there would be under microgravity when setting up the experiment.

*Specimen 1* was prepared as shown in Fig. 5a to determine whether nematodes reproduce, form the IJ stage, emerge, disperse, travel 10 cm in moist sandy soil, find a bait insect and infect/invade the host in microgravity (Fig. 1a–d). If the infection failed, this specimen was also designed to determine whether it was due to insect host-immune response. Specimen 1 was placed in cold stowage (−80 °C) 7 days after the launch at ISS, which was after the anticipated time of IJ invasion. A corresponding Earth control was prepared for both IJ invasion and insect host immunity response. Since we did not know how freezing or a month in −80 °C storage would affect hemocyte counts, we prepared additional Earth controls to be analyzed on the day the Earth Specimen 1 controls were placed in −80 °C (Supplementary Table 1). Three replicates were prepared for each treatment; microgravity, corresponding Earth control and additional Earth control for hemocyte counts. A total of 18 insects in nine individual tubes were analyzed for IJ invasion, to determine the IJ developmental stage and hemocyte counts for both Space and Earth specimens. The hemocyte counts were done for the additional Earth control (no freezing) on the day the other set was placed in the −80 °C freezer. Mean hemocyte count for the additional Earth control (no freezing) was 2.8 ± 1.7 from three replicates.

*Specimen 2* was prepared in the same manner as Specimen 1 except that the tubes were not frozen to allow IJs to develop and emerge from the bait insect. Unfortunately, they died upon return to Earth. Three replicates were prepared for each treatment; microgravity and Earth control with a total of six tubes analyzed.

*Specimen 3* was prepared the same as Specimen 1 except that it did not include a bait insect and the location of the infected insect was on the opposite side (Fig. 5). This specimen was designed to determine whether nematodes can continue their development and reproduction during the flight and emerge in microgravity. Since we did not know whether IJs could emerged from consumed cadavers under microgravity, we prepared Specimen 3. Sand was included to make sure that the emerged nematodes have a place to live until the experiment was returned to Earth and to make a good comparison to Specimen 1. Furthermore, Specimen 3 was kept at ambient temperature to test IJs dispersal after returning to Earth. Again, three replicate tubes for each treatment (microgravity and Earth control) were prepared with a total of six replicate tubes.

*Specimen 4* was prepared the same as specimen 1 except that it had 2000 IJs in 0.5 ml of polyacrylamide gel instead of an infected insect (Fig. 5a). This was a backup experiment just in case Specimens 1 and 2 failed to emerge in microgravity to infect bait insects. The column contained sand in the middle (causing a time delay) so the infection event would occur in space not on Earth. The experiment had to be delivered to NASA 36 h before the rocket launch. The sand provided enough time delay (2–3 days) that IJs would only find the bait insects when they reached the ISS or when the Dragon Capsule was in space. This experiment had an Earth control like the other experiments and one additional Earth control was included to determine whether the bait insects were infected before the rocket launch. Three replicates were prepared for each treatment; microgravity and two sets of Earth controls with a total of nine tubes analyzed.

The rocket launch was delayed 1 day from December 4 to 5. The IJs had 2 days, 21 h and 20 min to go through 10 cm sand to reach the bait insect as opposed to 1 day 21 h and 20 min as planned launch day December 4. This was very concerning. At the earliest time (1 and ½ h) after the rocket launch, we analyzed the additional Earth control (three replicates) for insect invasion in two steps. First, we placed the IJs in a petri dish and added water to rinse the IJs off the surface of the insects. Each replicate was analyzed on a separate petri dish. The bait insects were alive and in all three replicates had IJs on them. Next, we washed all the insects to remove all the IJs from their surface and looked inside the insects to determine whether any of the IJs were inside the insects. No IJs were found inside the bait insects suggesting that none of the insects were infected while they were on Earth.

*Specimen 5* contained *S. feltiae* IJs in polyacrylamide gel with a density of 4000 IJ/ml (Fig. 5b). A total volume of 10 ml of IJ acrylamide suspension was placed in each of three 15 ml conical tubes. This specimen was prepared to determine how 33 days of space flight would affect IJ adjustment (movement) and infectivity after returning to Earth. Upon return to Earth at the earliest time (3 days after returning), IJ movement was compared to an Earth control and 10 days later, IJ infectivity was tested. This was also a backup experiment in case nematodes died due to the effect of sand because IJs were known to safely travel on Earth in polyacrylamide gel^43^. Like the other experiments, three replicate tubes (120,000 IJs) for each treatment (microgravity and Earth control) were prepared with a total of six replicate tubes (240,000 IJs).

*Specimen 6* had two *G. mellonella* last instar larvae in 15 ml conical tubes in wood shaving (Fig. 5c). This was to determine if the infection failed in Specimen 1 due negative effects on the insect host. At the end of the space flight, insects in both microgravity and Earth control were dead at different developmental stages. Several insect stages were observed in the various tubes (larvae, pupae and adult), all of which died. A total of 12 insect larvae in six replicate tubes were analyzed.

All Specimens, except for Specimen 1, were kept at ambient temperature on the ISS, during the flight back to Earth, and for 3 days while being shipped to our laboratory for further analysis on nematode IJ fitness and infectivity.

### Handing over the experiments to NanoRacks for delivery to NASA for launch

Specimens 1–4 and 6 in Fig. 5 were assembled at Kennedy Space Center (KSC) Space Station Processing Facility (SSPF) on December 2 (Supplementary Table 1). Specimen 5 in Fig. 5b was prepared on Nov 29 at the Shapiro lab in Byron, GA, and kept at 5 °C until December 2. After all of the specimens were prepared (Supplementary Fig. 1), Specimen 1 replications were placed in a sealed bag and Specimens 2–6 were placed in a separate sealed bag. They are weighed and placed in Nanotracks’ NanoLab in a Horizontal form (Supplementary Fig. 1). The Nanolab lid was sealed with Velcro. After that, the Nanolab was delivered to NASA and placed in the Dragon Capsule on SpaceX Falcon9 rocket for CRS-19 on the same day (Supplementary Table 1). The experiments in the Nanolab were placed in a horizontal position and kept 20 °C for 2 days, 21 h and 20 min in the Dragon capsule until the launch on December 5, 2019. Experiments were kept in a horizontal position during the launch, in microgravity in Space, and at ISS at ambient temperature until returned to Earth.

Earth controls: Specimen 1 for Earth control was kept in a sealed plastic bag and Specimens 2–6 were placed in a separate sealed plastic bag at ambient temperature at the KSC SSPF until launch on December 5. Specimen 1 Earth controls were taken to the Shapiro Lab by car and kept at RT until December 11. Specimens 2–6 were shipped by FedEx at ambient temperature using cool packs for maintenance of the temperature to the Pheronym R&D laboratory, Davis, CA

### In orbit and return to Earth

Specimen 1 (the three 15 ml conical tubes) was transferred to cold storage on December 12 at 346/14:30 (8:30am CST) by European Space Agency Astronaut Luca Parmitano (https://blogs.nasa.gov/stationreport/2019/12/12/). Specimens 2–6 were kept in the NanoLab on the Dragon Capsule which returned to Earth on January 7, 2020 and was retrieved from the Pacific Ocean by Space X. NanoRacks received the samples on Jan 9, 2020, in their facility in Houston, TX and shipped Specimen 1 (kept frozen) in dry ice overnight by FedEx to the Shapiro Lab in Byron, GA. The Shapiro Lab in Byron GA placed the samples in −80 °C until analysis. On the same day (Jan 9), NanoRacks shipped Specimens 2–6 at RT overnight by FedEx to Pheronym, Davis, CA. On January 10, 2020 at 10 AM, Specimens 2–6 were received by Pheronym Lab, and were inspected immediately for dead, alive sluggish or active nematodes, pictures were taken, and videos recorded before removing the Teflon seals. Pictures were taken using a Nikon D60 (Nikon, Tokyo, Japan). Videos were taken with a hand-held USB Celestron microscope (Celestron, Torrance, CA) and/or iPhone 6 S (Apple, Cupertino, CA).

### Immune response assessment of Specimen 1

The primary immune defense in insects against multicellular parasites including EPNs is encapsulation. Immune cells (hemocytes) bind to the nematode and one another to form a multicellular envelope; we therefore assessed immune response based on hemocyte activity and encapsulation^46^. *G. mellonella* larvae were dissected longitudinally. The presence of hemocytes adhering to the nematode body was confirmed at 400–600X. The number of nematodes showing hemocyte activity was compared between treatments.

### Assessing the IJs after returning to Earth

When Specimen 2, 3, 4, and 5 were received, *S. feltiae* IJs were observed visually to assess whether they were alive, sluggish, or dead before removing the seals. Microgravity and corresponding Earth controls were inspected at the same time. There were several IJs visible prior to opening the tubes. Subsequently, we unsealed the tubes for Specimen 2, 3, and 4, removed half of the sand to a 10 cm petri dish and harvested the IJs by washing the sand three times with water. The IJs were then rinsed with MILLIQ water to test for dispersal behavior at Pheronym and sent to the Shapiro-Ilan’s lab for assessing symbiotic bacteria load. In case we missed any live IJs in Specimens 2, 3, and 4, we baited the other half of the sand with two *G. mellonella* larvae and incubated them at 22 ± 1 °C for three days before inspecting for dead or live insects. After that the samples were shipped to the Shapiro-Ilan’s lab for a second inspection and analysis of the bait insects for IJ presence.

Specimen 5 was in polyacrylamide gel which was transparent, making visually scoring easy dead or alive and taking videos (Supplementary Video 1 and 2). After inspection, three ml of IJs with a density of 4000 IJs/ml were shipped in gel at ambient temperature overnight by FedEx to the Shapiro-Ilan lab to conduct infectivity experiments.

### Dispersal assays of Specimens 2 and 3

Dispersal assays were conducted as described by Kaplan et al.^14,15^ with a few differences. Petri dishes (6 cm) containing 0.9% agar with a gel strength: ≥900 g/cm^2^ (Caisson Agar, Type I, Smithfield, UT) were used for the assays. Briefly, IJs from Specimens 2 and 3 were tested for dispersal immediately by rinsing three times with MILLIQ water after the harvest from sand. The assays were conducted in the afternoon between 2 and 3 PM. Approximately ~26–55 IJs (due to a limited number of IJs) in 10 µL of water were placed in the center of an agar plate with 6 cm diameter Petri dishes and counted. The assays were run for 30 min during which IJs were free to move on agar plates. After 30 min, IJs inside and outside the 1.3 cm ring were counted. IJs remaining inside the 1.3 cm ring were considered non-dispersed, and those outside were considered to have dispersed. Percentage dispersal was calculated as the number dispersed relative to the total number of IJs on the plate. Three replications for Specimens 2 (proxy Earth control) and Specimen 3 (treatment, microgravity), a total of six plates were analyzed.

### Infectivity after 33-day space flight, Specimen 5

Infectivity was assessed based on procedures describe by Mbata et al.^46^. IJs were extracted from gel by dilution and centrifuged at 582*g* to concentrate them. A total of 960 IJs per replication were pipetted onto filter paper (Whatman No. 1) in a 0.35 mm Petri dish. A single greater wax moth, *Galleria mellonella* (L.) larva was added to each Petri dish. Insects were incubated in the dark for at 25 °C for 72 h and then dissected under a stereomicroscope to determine the number of invading IJs. There were five insects per replicate of Specimen 5 (total six replicates microgravity and Earth controls) and insect only (no IJs) control. A total of 35 insects were analyzed.

### Comparison of symbiotic bacterial load per nematode in Specimens 2 and 3

Methods to compare symbiotic nematode bacteria load among treatments were based on those described by Kaya and Stock^1,5^. IJs were surface sterilized with 0.5% NaClO and then washed three times with sterile distilled water (centrifuging at 582*g* for 2 min between each wash). The final pellet was suspended in 0.5 ml. Single IJs were homogenized for 60 s with a sterile motor-driven polypropylene pestle and then transferred onto 60 mm nutrient agar plates. The plates were incubated at 25 °C. The number of bacterial colonies per IJ was assessed at 3 and 7 days.

### Statistical analysis

For comparisons of infectivity, dispersal behavior, symbiotic bacteria load, and immune response the treatments effects (from space) were compared to Earth controls using Student’s *t* tests (SAS version 9.4, SAS 2002). Based on the inspection of residual plots, numerical data were log transformed prior to analysis^47^ (SAS, 2002).

### Ethics statement

We use the invertebrate model system *Steinernema feltiae* and *Galleria mellonella* for this study, in accordance, the study was exempt from ethics committee approval.

### Reporting summary

Further information on research design is available in the Nature Research Reporting Summary linked to this article.

## Supplementary information

Supplementary Video 1

Supplementary Video 2

Reporting Summary

Supplemental material

## Data Availability

All data are available in the main text or the supplementary materials.
