# Supplementary material for: Dynamics of entomopathogenic nematode foraging and infectivity in microgravity
Source: NPJ Microgravity. 2020 Aug 10;6:20. doi: 10.1038/s41526-020-00110-y (PMC7418002; doi:10.1038/s41526-020-00110-y)
Supplement: Supplementary file 4 — Supplemental material [file 41526_2020_110_MOESM4_ESM.pdf]

Supplementary information

Dynamics of entomopathogenic nematode foraging and infectivity in microgravity

Fatma Kaplan[1*], David Shapiro-Ilan[2*], Karl C. Schiller[1]

[1]Pheronym, Inc. Davis, CA, 95618, USA
[2]US Department of Agriculture, Agricultural Research Service, Byron, GA 31008, USA

*Correspondence to: fkaplan@pheronym.com, david.shapiro@usda.gov.

Supplementary Figure

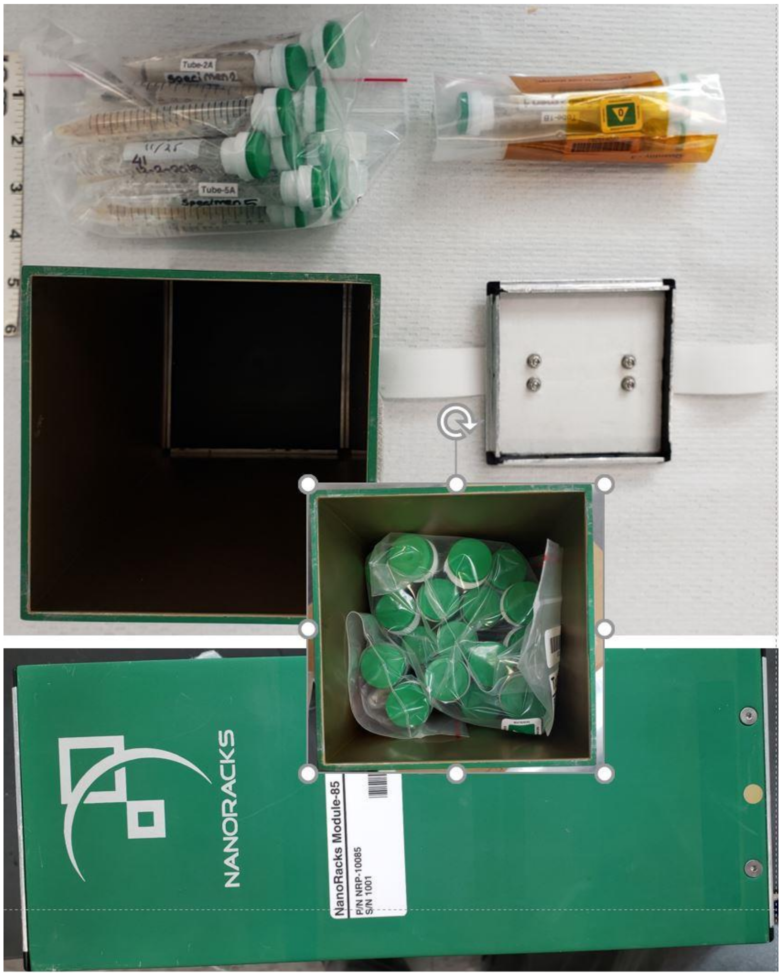

Supplementary Figure 1. *Steinernema feltiae* agriculture biocontrol experiments in NanoRacks Module-85. Specimens were incubated at the NanoRacks Module-85 during the microgravity experiment from December 2, 2019 to January 10, 2020. Module-85 was placed in CRS-19 SpaceX Dragon capsule and ensured that during the flight to ISS, after and in microgravity the specimens were in a horizontal position to prevent potential injury to bait insects or rupture to infected insects

Supplementary Table

Supplementary Table 1. Timeline of activities for *Steinernema feltiae* biocontrol experiment in microgravity

	Date and time	Activity	Additional information
**Pre-Launch**	**Jan 18 - Dec 5, 2019**		
	Jan 18 - Oct 15	1- NASA Phase 0/I/II/III safety certification, 2- Scheduling launch date, 3- Payload turnover date, 4- Flight configuration experiments, 5- Ground testing	NanoRacks, LLC., Pheronym, Inc. and ISS US NL
	Nov 11 - Dec 5	Logistics and preparations for Specimens 1-6	Pheronym, Inc., and USDA-ARS
	Nov 13-14	*G. mellonella* larvae were infected with *S. feltiae* IJs.	
	28-Nov	*S. feltiae* IJs were harvested, rinsed, and placed in polyacrylamide gel for Specimen 5, which was placed in 5 degree Celsius	Live organisms were driven to **Kennedy Space Center (KSC) Space Station Processing Facility (SSPF)** by Shapiro-Ilan Lab to avoid stress due to air travel (potential pressure change) for *S. feltiae* IJs and insect hosts. Non-living materials were prepared and shipped to KSC by Pheronym, Inc.
	**29-Nov**	G. mellonella larvae were infected to prepare for Specimens 1-3.	
	30-Nov	All the materials living and non-living arrived at Merritt Island, FL.	
	**2-Dec**	Times are in EST	
	**10 AM 3 PM**	Specimens 1-4 and 6 were prepared. Specimen 5 was brought to room temperature	
	3:00 PM	Specimen 1-6 in the NanoLab were handed over to NanoRacks	
	4:39 PM	Specimens 1-6 in were handed over to NASA and approved for launch	
	Dec 4	Launch scrubbed	
	Dec 2-5	Experiments (Specimens 1-6) in NanoRacks Module-35 were kept at RT in SpaceX Dragon Capsule until the CRS-19 launch.	
**Launch**	**Dec 5, 2019**		
	12:29 PM	SpaceX Falcon9 Dragon capsule launched	The launch was scheduled for Dec 4, 2019. It was delayed due to the low temperature in the upper atmosphere
	**12:39 PM**	Experiment is Space (in microgravity)	
**Post-Launch**	**Dec 5, 2019**		

		2:09 PM	Specimen 4 additional Earth control (3 replications) was analyzed at KSC-SSPF	This was done to determine whether IJs reached the bait insects and invaded the host before the experiment reached space.
	Dec 6		Earth control for Specimen 1 were kept at RT and driven to the Shapiro-Ilan lab. Earth controls for Specimens 2-6 were kept at RT and shipped by FedEx to Pheronym, Davis CA.	
	Dec 8	5:05 AM	Experiments in SpaceX Dragon capsule reached The ISS US NL	The station commander, Italy's Luca Parmitano, used a large robot arm to grab onto the Dragon, which soared 260 miles above the South Pacific at the time of capture.
	Dec 11		Specimen 1 Earth control (3 Replications) was placed in -80C	The additional Specimen 1 Earth controls (3 reps) were analyzed for IJ invasion and hemocyte counts without freezing
**In orbit**	**Dec 12**			
	Dec 12	9:30 AM	Specimen 1 was placed in cold stowage (-80C) at the ISS US NL until return to Earth	
**Return to Earth**				
	**Jan 7, 2020**			
		6:05 AM	SpaceX Dragon capsule was released from ISS	
		11:40 AM	Dragon capsule splashdown in the Pacific Ocean southwest of Long Beach, CA	
	Jan 9		Specimen 1 was shipped frozen in dry ice to the Shapiro-Ilan Lab at the USDA, Byron GA. Specimens 2-6 were shipped at RT to Pheronym, Davis, CA.	NanoRacks shipped the experiments on the same day they were received.
	Jan 10	9:00 AM	Specimen 1 was received and placed in -80C until analysis	USDA-ARS
		1:00 PM	Specimen 2-6 were received. The initial analysis was conducted on Specimens 2-6 and their corresponding Earth controls.	Pheronym, Inc., received the samples at 10 AM Pacific time which is 1 PM Eastern time.
	Jan 12		IJs from Specimens 2, 3 and 4 were harvested from the sand. We conducted agar plate dispersal assays using the IJs.	

Jan 13	Specimen 1 was analyzed for IJ invasion and host immune response
Jan 14	Specimens 2-5 were sent to the Shapiro-Ilan lab overnight by FedEx for further analysis or confirmation.
Jan 17	Infectivity analysis with Specimen 5

Supplementary Videos

Supplementary Video 1. Activity of *Steinernema feltiae* IJs in Specimen 5 after 33-day space travel. Movie is taken 3 days after returning to Earth. Movie is a representative of the 3 replications.

Supplementary Video 2. Earth control of *Steinernema feltiae* IJs in Specimen 5. Movie is a representative of the 3 replications.
